# N6-Methyladenosine in RNA and DNA: An Epitranscriptomic and Epigenetic Player Implicated in Determination of Stem Cell Fate

**DOI:** 10.1155/2018/3256524

**Published:** 2018-10-10

**Authors:** Pengfei Ji, Xia Wang, Nina Xie, Yujing Li

**Affiliations:** ^1^Department of Nutrition and Food Sciences, Texas A&M University, Room 218 Cater-Mattil, MS 2253 College Station, TX 77840, USA; ^2^Department of Dermatology, Rizhao People's Hospital, Rizhao, Shandong Province 276826, China; ^3^Department of Neurology of Xiangya Hospital, Central South University, Changsha, Hunan 410008, China; ^4^Department of Human Genetics, Emory University School of Medicine, 615 Michael St., Atlanta, GA 30322, USA

## Abstract

Vast emerging evidences are linking the base modifications and determination of stem cell fate such as proliferation and differentiation. Among the base modification markers extensively studied, 5-methylcytosine (5-mC) and its oxidative derivatives (5-hydroxymethylcytosine (5-hmC), 5-formylcytosine (5-fC), and 5-carboxylcytosine (5-caC)) dynamically occur in DNA and RNA and have been acknowledged as important epigenetic markers involved in regulation of cellular biological processes. N6-Methyladenosine modification in DNA (m6dA), mRNA (m6A), tRNA, and other noncoding RNAs has been defined as another important epigenetic and epitranscriptomic marker in eukaryotes in recent years. The mRNA m6A modification has been characterized biochemically, molecularly, and phenotypically, including elucidation of its methyltransferase complexes (m6A writer), demethylases (m6A eraser), and direct interaction proteins (readers), while limited information on the DNA m6dA is available. The levels and the landscapes of m6A in the epitranscriptomes and epigenomes are precisely and dynamically regulated by the fine-tuned coordination of the writers and erasers in accordance with stages of the growth, development, and reproduction as naturally programmed during the lifespan. Additionally, progress has been made in appreciation of the link between aberrant m6A modification in stem cells and diseases, like cancers and neurodegenerative disorders. These achievements are inspiring scientists to further uncover the epigenetic mechanisms for stem cell development and to dissect pathogenesis of the multiple diseases conferred by development aberration of the stem cells. This review article will highlight the research advances in the role of m6A methylation modifications of DNA and RNA in the regulation of stem cell and genesis of the closely related disorders. Additionally, this article will also address the research directions in the future.

## 1. Introduction

Epigenetics is defined as the gene expression alterations heritable to next generations caused by nongenetic but heritable cellular memory other than DNA sequence variations [1]. The epigenetic memories including dynamic base modifications (DNA methylation/demethylation), histone modifications, chromatin architecture, and noncoding RNAs maintain all the biological processes in the programmed tracks. It is true that a microevent in base modifications could lead to strong “earthquake” in metabolic pathways and the consequent alteration of organism phenotypes. Therefore, any aberrant alterations could lead to development of abnormality and initiation of diseases such as neurological disorders and cancers as reviewed in [2–8].

DNA base modifications such as methylation of 5-cytosine (5-mC) [9–14] and 5-hydroxymethylcytosine (5-hmC) [15–21] have been acknowledged as the best characterized epigenetic markers in mammalian brains [22–25] and ES cells [26–28], essentially regulating chromatin structure and consequently gene expression with the potential mechanisms. In the present review article, we highlight advances in another base modification N6-methyladenine which exists in both DNA (m6dA) and RNA (m6A) and is not new in terms of its discovery history, but its biological functions are being gradually unveiled only in recent years in regulation of the development and stem cell fate. Meanwhile, the future research directions in N6-methyladenine are addressed as well.

### 1.1. RNA m6A Modification

Fine-tuning functions and metabolic regulation require posttranscriptional modifications of RNA transcripts. Among more than 100 of the chemical modifications in RNA from almost all the known living organisms [29–31], N6-methyladenosine (m6A) has been recognized as the most abundant in quantity and prominent in its power and range of the regulation functions in eukaryotic mRNA, leading to the significant efforts paid particularly in recent years with invention and application of high-throughput sequencing as well as advances in modern molecular and genetic technologies.

**Figure 1:**
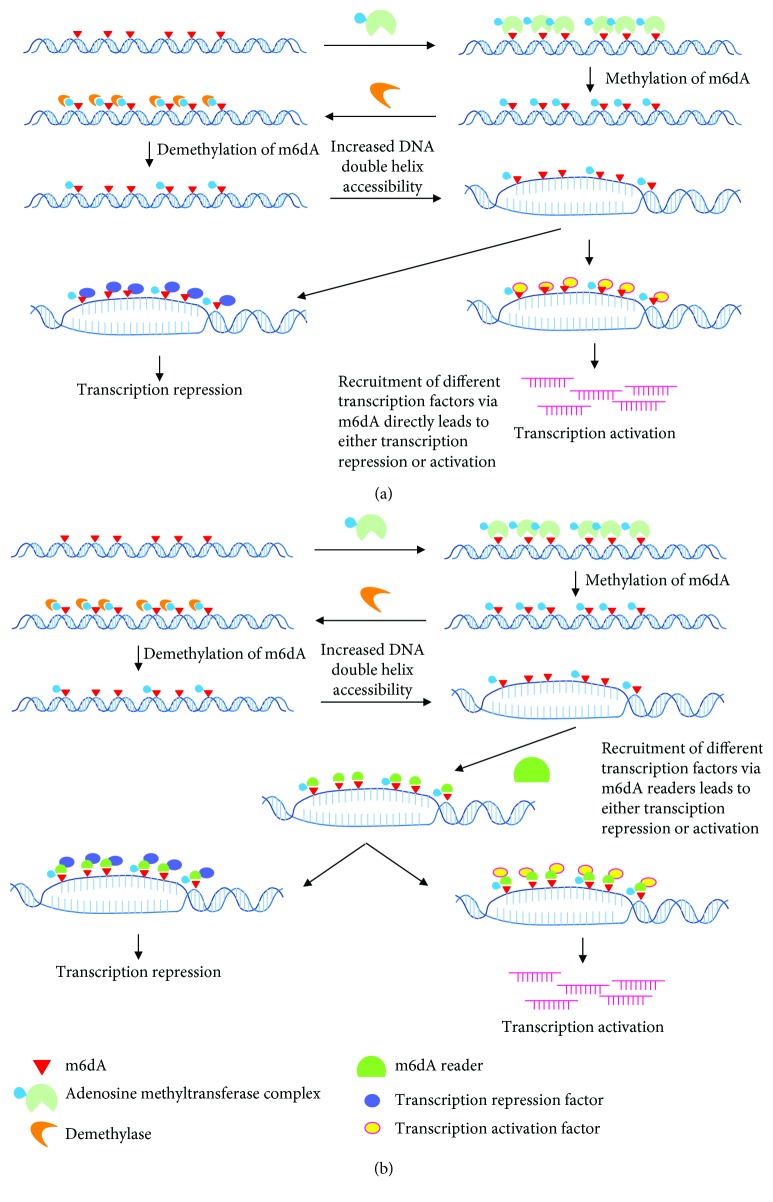
Dynamic regulation of genomic DNA N6-methyladenosine (m6dA) levels by unknown/known components and the potential functions of m6dA in the regulation of gene expression. The coordination between m6dA writer(s) and m6dA erasers maintains the m6dA levels in accordance with physiological conditions and the development and growth stages. (a) The hypothesis for m6dA-mediated regulation of gene expression is that by decreasing the binding energies of base pairs, m6dA could destabilize the DNA duplexes, facilitating m6dA-enriched regions of DNA, unwinding, or making the DNA structure more open for transcription initiation. The m6dA readers (to be identified) are highly affinitive to and bind to the m6dA sites, then the readers may recruit their interaction factors involved in transcription initiation, repression, and so on. (b) Alternatively, it is possible though that these transcription factors might serve as reader(s) of m6dA, directly functioning as regulators of gene expression.

**Figure 2:**
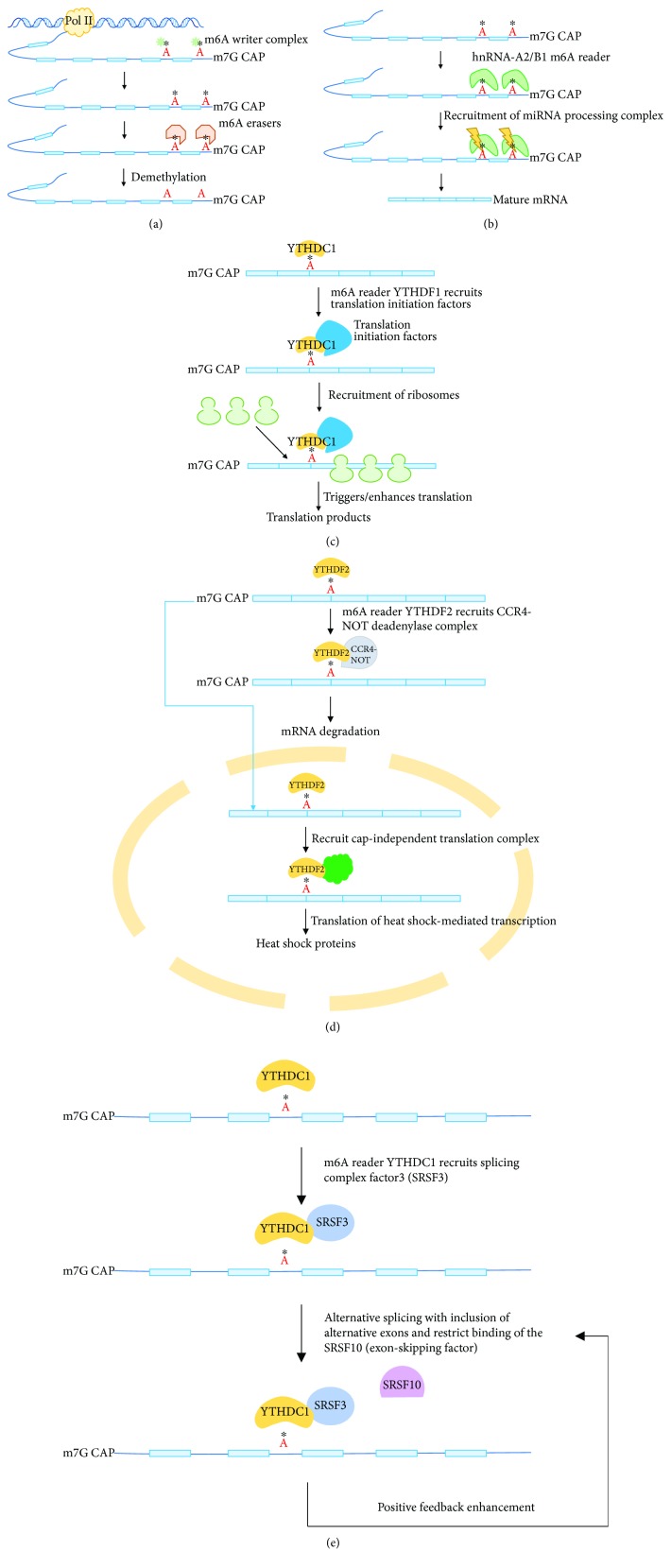
Dynamic regulation of RNA m6A levels by the m6A processing machinery and the known functions of m6A in regulation of RNA metabolism. (a) The coordination between m6A writers and m6A erasers maintains the m6A levels in accordance with the physiological conditions and the development and growth stages. (b) m6A reader hnRNP-A2/B1 mediated microRNA processing. The hypothesis for m6dA-mediated regulation of gene expression is that m6dA readers (to be identified) are highly affinitive to and bind to the m6dA sites, then the readers may recruit their interaction factors involved in transcription initiation, repression, and so on. (c) Via binding to m6A sites to recruit the translation initiation factors, m6A reader YTHDF1 triggers initiation of translation and releases the RNA transcripts to the ribosomes. (d) By recruiting the CCR4-NOT deadenylase complex after binding to m6A sites, the reader YTHDF2 could enhance mRNA decay. On the other hand, facing heat shock, YTHDF2 could transport to the nuclei to trigger the cap-independent translation to translate the heat shock-related RNA transcripts into heat shock proteins. (e) YTHDC1 binds to the m6A sites on the pri-mRNA transcripts and recruit splicing complex factor 3 (SRSF3) to trigger alternative splicing with inclusion of alternative exons. Meanwhile, by recruiting SRSF3, YTHDC1 could restrict binding of SRSF10, further enhancing alternative splicing.

RNA m6A is catalyzed by a multicomponent methyltransferase complex (the “writer”), preferentially bound by binding proteins (the “readers”), and could be removed by specific demethylases (the “erasers”) (Figures 1 and 2, Table 1). Recent studies on mRNA m6A modification have linked the m6A-dependent control of mRNA homeostasis to posttranscriptional regulation of gene expression involved in a wide spectrum of metabolic pathways, consequently.

### 1.2. DNA N6-Adenosine Modification (m6dA)

N6-Methyladenine modification is not only a RNA marker (m6A) but also a genomic DNA marker (m6dA). The initial discovery of m6dA was from prokaryotes [32] particularly in bacteria, but later on it was detected in lower eukaryotes as well [33–43]. In higher eukaryotes, alteration of m6dA levels from the most abundant during embryogenesis to the significant decrease in adult tissues suggests its importance for development and a potential link with regeneration maintenance. Like 5-hmC loss as a hallmark for cancer cells, a significant decrease in m6dA levels has also been reported in a variety of cancer cells (unpublished data).

## 2. Distribution of m6A and m6dA in Epitranscriptomes and Epigenomes

### 2.1. m6A Distribution in Epitranscriptomes

The sequencing data from mRNAs of several organisms indicated that m6A-methylated mRNA accounts for only ~25% of the total cellular mRNA, suggesting the high selectivity and specificity of m6A sites in the target mRNAs although related mechanisms remain to be elusive.

The m6A distribution was nonrandom and asymmetric in a way that majority of m6A sites were highly enriched within 5′UTR, 3′UTR, stop codon, and long introns relative to the coding region (Table 2) [44, 45]. In addition, the m6A landscape is dynamically altered in accordance with the development stages and physiological conditions, but highly conserved among the mammal species at the corresponding conditions, indicating the regulation of the development and the significant functional relevance [44]. However, some studies argue that m6A functions as an even faster approach to posttranscriptionally enhance gene expression [46]. Additionally, m6A is believed to have a special function during developmental transitions by leading the m6A-marked transcripts to degradation [46].

### 2.2. m6dA Distribution in Epigenomes

#### 2.2.1. m6dA Distribution in the Genomes of Eukaryotes

The genome-wide distribution of m6dA in genome has been identified and characterized by using multiple strategies ([42, 47], Yao et al., unpublished data [42, 48–51]). However, so far none of these methods alone could offer the accurate detection of m6dA distribution in the genomes, implicating the indispensable multiple strategy-based cross-validation for the high efficiency and sensitivity.

m6dA-IP-seq, SMRT-seq, and single-molecule long-read-seq have contributed significantly to identification of genomic loci of m6dA in genomes of *C. elegans* [39], *Drosophila* [40], *Chlamydomonas* [36], and fungi [52] as summarized in Table 2. Unlike the distribution of the m6A sites in the epitranscriptomes, the distribution of m6dA greatly varies from genome to genome. Using single-molecule long-read-seq, m6dA levels and genomic distribution were compared in 16 diverse fungal genomes. It turns out that the ratios of m6dA to all adenine bases (A) reach up to 2.8%; dramatically higher levels than all other eukaryotes so far have been identified [52]. 80–99.6% of the m6dA sites among the diverse genomes were located at the AT motif symmetrically and significantly enriched in the heavily methylated m6dA clusters near the downstream TSSs of the actively expressed gene promoters [52]. More interestingly, m6dA distribution was inversely correlated with abundance of 5-mC.

While in *C. elegans* m6dA showed no region preference across the genome, it mainly distributed in transposon elements as well as in CNS in fly genome ([40, 42], and unpublished data). Particularly, our group found a large percentage of 6mA on intragenic regions with particular enrichment in introns and untranslated regions (UTRs) in *Drosophila* neuron cells BG3C2 (Yao et al., unpublished data). By contrast, m6dA is preferentially enriched at transcription start sites (TSSs), in promoter, genic, and intergenic regions [53], and in the nucleosome-linker DNA with an A-T sequence motif in *Chlamydomonas* [36, 53].

SMRT-seq based m6dA mapping in the genome of *Tetrahymena* indicated that m6dA is enriched at the 5′ end of the gene body and AT motif of the linker DNA regions flanked by nucleosomes particularly H2A.Z- (a variant of the H2A) containing nucleosomes [54]. In addition, m6dA is specifically associated with Pol II-transcribed genes, altogether suggesting that m6dA serves as an indispensable component of the chromatin landscape, playing a part in chromatin remodeling and gene expression at the transcription level.

In the mouse brain, m6dA was substantially biased in its genomic distribution, depending on the gain or loss of m6dA in accordance with stress or normal physiological conditions. The gain of m6dA upon stress is highly enriched in the intergenic regions of the prefrontal cortex (PFC), while it intragenically associated with introns and is excluded from most coding exons [42]. The SMRT-ChIP-seq-based assays have identified the significant enrichment of m6dA in deposition regions of H2A.X (a H2A variant) and at intergenic but not at gene-rich regions as well as at transposon LINE-1 in mouse and mouse ES cells [54]. The motif diversity and nonrandom distribution of m6dA in distant genomes suggest the potentially biological functions unique to a specific organism.

## 3. Methyltransferases (Writers) for m6A Methylation in RNA and DNA

### 3.1. Writers of RNA m6A in Eukaryotes

A multiple component complex consisting of heterodimer of METTL3–METTL14 linked with WTAP and KIAA1429 has been characterized as main writers to methylate base adenosine in the conserved region ACU [55–60]. The different components in the complex have been specified for their individual roles and work together concordantly to carry out their functions more efficiently from recognition and precise localization of the m6A methylation sites to methylate the adenosine sites to m6A. Knockout or knockdown of either Mettl3 or Mettl14 led to depletion or dramatic decrease in the m6A levels in RNA, suggesting their function as methylase for RNA m6A methylation [58, 59, 61].

Although METTL3 has been acknowledged as the main methyltransferase, more and more components in the methyltransferase complex are being identified, such as ZFP217, RMB15, and RMB15B binding to the specific target sites of the RNA to execute specific functions. WTAP is believed to be responsible for recruiting the METTL3–METTL14 complex to nuclear speckles [57, 58] where RNA adenosine methylation occurs. METTL14, the partner of METTL3, though no methyltransferase activity was detected, could facilitate RNA methylation site recognition [62]. Additionally, RNA-binding motif protein 15 (RMB15) and its paralogue RMB15B recruits the METTL3-WTAP complex to m6A consensus sites for methylation [63].

### 3.2. Writers of DNA m6dA in Eukaryotes

Any base modification can be dynamically regulated in accordance with stages of growth, development, and reproduction, including generation by writers and removal by erasers. While methyltransferases and demethylases for m6A RNA modification have been identified and well characterized, so far only limited information is available for the methylation and demethylation of DNA m6dA modification. The main methyltransferases for RNA m6A such as the complex consisting of METTL3/METTL14 [62, 64–66] have only weak activity in DNA methylation in humans [58]. In other well-characterized DNA methyltransferases for 5-cytosine methylation such as DNMT family members, so far no evidences show their activity to transfer the methyl group to the 6dA base to generate m6dA. Likewise, for N6-methyladenosine transferases for the formation of RNA m6A, such as the IME4 inducer in *Saccharomyces cerevisiae* [67, 68], the MT-A70 domain in humans [69], and DAMT-1 in *C. elegance* [39], so far there are no direct biochemical evidences to show whether they really function as genomic DNA methyltransferases as well. Collectively, it seems that the majority of methyltransferases for N6-adenosine methylation in RNA have either weak or no activity at all for genomic DNA adenine methylation, indicating that although it is completely the same base methylation event, few crosstalks occur between the event in DNA and that in RNA.

## 4. Readers of RNA m6A and DNA m6dA

### 4.1. Readers/Effectors of RNA m6A

Transformation from the epitranscriptomic information engraved in RNA m6A to functional signals is carried out by a special class of proteins defined as m6A readers or effectors. The readers/effectors are highly affinitive to the m6A sites due to alteration of the secondary or tertiary structure in specific domain(s) of the target RNAs where the m6A sites were disposed. Since none of the known m6A readers were confirmed to be directly involved in miRNA biogenesis, mRNA maturation, splicing factors, or mRNA half-life, functions of the m6A marker are most probably executed by the m6A reader-mediated downstream events (Figure 2). By binding to the m6A surrounding domain(s), the m6A readers/effectors could alter the RNA-protein conformation to pave the way for recruitment of the second protein component either by direct interaction with readers or by binding to the new site(s) created during the protein-RNA conformation remodeling. The recruitment of the second protein may determine the fate of the target RNA as the recruited proteins were referred to be involved in mRNA metabolism. So far, a category of m6A reader protein components has been identified and these components are classified as several families, including the YTH domain [70–82], hnRNP family including hnRNP-A2/B1, hnRNP-C, hnRNP-G, hnRNP-F, hnRNP-H1, and hnRNP-H2 [83–87], KH domain, zf-CCHC domain, RBD, RRM, and zinc knuckle domain protein families [88–92], as summarized in Table 1.

### 4.2. RNA m6A Repellers

In addition to the m6A readers, the m6A repel proteins (or m6A repeller) were identified as well in a recent study [87]. The m6A repellers preferentially interact with an unmodified RNA sequence but repelled by m6A, such as G3BP1 and G3BP2 known as stress granule proteins [93, 94], USP10 and CAPRINI (interaction partners of G3BP1 and G3BP2), and METTL16, an adenosine methyltransferase for small nuclear RNA. Compared to the m6A readers, these repellers were more diverse and cell type-dependent [87]. It has been confirmed both in vivo and in vitro that the RNA m6A repellers positively affect the stability of the target mRNAs by binding to their mRNA targets [87].

## 5. Functions of RNA m6A and m6dA Erasers

### 5.1. RNA m6A Erasers

Adenosine methyltransferases and demethylases (erasers) concordantly work together to regulate dynamic levels of m6A and the landscapes during the stages of generation, development, and reproduction. The functional study on m6A has been lagged behind until the discovery of its erasures in recent years [85, 95, 96]. So far, several demethylases have been identified and characterized for both DNA m6dA and RNA m6A.

#### 5.1.1. FTO

So far, only two members have been identified to exhibit the comparable demethylase activity, including FTO and ALKBH5 [77, 95–97]. FTO, belonging to the AlkB family of non-heme Fe (II)/a-ketoglutarate- (a-KG-) dependent dioxygenases, was the first demethylase identified to demethylate m6A in RNA [96]. FTO is mainly expressed in the brain and adipose tissue [95, 98, 99]. More specifically, like TET proteins that convert 5-mC to 5-hmC, 5-fC, and 5-caC, FTO could oxidize m6A to its intermediate form N6-hydroxymethyladenosine (h-m6A) and N6-formyladenosine (f-m6A). However, these intermediates' functions remain to be elusive whether they are just intermediates with short lifespans to be finally converted to regular adenosine or they serve as special modification markers to further affect RNA-protein interactions [100].

#### 5.1.2. ALKBH5

Four of the nine *E. coli* AlkB family homologs in mammalian (ALKBH1-9) cells have been characterized as diverse demethylases functioning as removal of the methyl group from ribonucleobases, including ALKBH1, ALKBH5, ALKBH8, and ALKBH9, respectively [85, 95, 101]. Next to FTO, ALKB5 was the second demethylase identified to erase the methyl group of m6A in eukaryotic RNA, regulating mRNA export and RNA metabolism as well as fertility in mammals phenotypically. In contrast to FTO with preferential expression in the brain and adipose tissues [98, 99], ALKBH5 is highly expressed in testes [95], suggesting that the tissue-preferential expression of demethylase is responsible for local demethylation activity. Disorders of the ALKB family level in mammals induce many types of diseases, suggesting the essential roles of the dynamic m6A levels in the life process.

More recently, DDX3, a member of DEAD box RNA helicases, was found to interact with ALKBH5 through its ATP domain and DSBH domain of ALKBH5 to modulate mRNA demethylation activity. Moreover, DDX3 regulated the m6A methylation status of microRNAs. This result suggests that the potential partners for demethylases such as DDX3 could regulate the demethylase activity more efficiently and precisely [102].

### 5.2. DNA m6dA Erasers

As for demethylation, although 5-mC could be demethylated by ten eleven translocation protein (TET) family members in eukaryotic genomic DNA particularly in mammals [103], it seems that majority of these members are not functional for m6A demethylation in RNA. Likewise, the identified majority of enzymes for demethylation of RNA 6mA such as ALKBH5, one member of the AlkB family of dioxygenases [95], showed very weak or even no activity at all for m6dA in DNA. However, FTO has been identified to catalyze demethylation of m6dA in synthetic DNA [96] and an even stronger activity than in RNA m6A demethylation under in vitro conditions, but still lacks evidence if it works for genomic DNA in vivo.

#### 5.2.1. DMAD

The homologue of the mammalian ten eleven translocation protein family (TET) [104] is the first demethylase for erasure of m6dA in DNA identified in *Drosophila* ([40], Yao et al., unpublished data). DMAD belongs to the TET protein superfamily, which functions in demethylation of 5-mC in mammals, but so far no report is available for mammalian TETs that could catalyze the demethylation of 5-mC to 5-hmC. A histone H3K4me2 demethylase SPR-5, a potential m6dA demethylase in *C. elegans*, could function as a putative m6dA demethylase as the SPR-5 deficiency mutant elevates the level of m6dA transgenerationally [39], but further biochemical evidences are required to support the conclusion.

#### 5.2.2. ALKBH1

The second demethylase has been characterized in mammalian ES cells to catalyze the demethylation of the DNA m6dA [47, 105, 106]. So far, it is not clear if this demethylase functions as demethylation of m6A in RNA.

#### 5.2.3. FTO

Under in vitro conditions, the first identified demethylase FTO for RNA m6A also shows an even stronger activity for demethylation of synthetic m6dA in DNA strands than that in RNA strands [96], suggesting the potentially strong demethylase of m6dA in genomic DNA. Later on, Huang et al. [107] further confirmed an inverse correlation between FTO expression and the m6dA levels in genomic DNA, suggesting that FTO functioned as a DNA m6dA eraser although in vitro biochemistry evidence is still not available.

## 6. Regulation Functions of RNA m6A

Even though significant efforts have been made in the study on RNA m6A modification, precise regulation mechanisms remain largely unknown. However, emerging evidences suggest that RNA m6A modification is indispensably involved in a wide range of spectrum of biological functions at both molecular and phenotypical levels. At molecular levels, m6A regulates RNA metabolism, including mRNA [56, 61, 74, 80, 83, 84, 95, 108–116], rRNA, tRNA, miRNA [83, 115], and circRNA [116].

### 6.1. For mRNA

m6A modification regulates mRNA stability [56, 61, 74, 108, 117], clearance [75], alternative splicing [80, 109–111], transportation and localization [95], translation efficiency [112, 113], and mRNA-protein interactions [84, 114].

### 6.2. Reciprocal Regulation of miRNA Maturation and m6A Methylation

hnRNP protein family members such as hRNPA2/B1 and hnRNP-C serve as m6A readers. hnRNP-A2/B1 shows high affinity to m6A that was methylated by METTL3 and located in pri-miRNAs. After binding to m6A, hRN-A2/B1 recruits the microprocessor complex to the miRNA precursors, enhancing processing of the precursors into mature miRNAs [83, 115]. Reciprocally, by base priming with their specific target mRNA sequences, miRNAs regulate the m6A modification level via repression of the binding of METTL3 to mRNAs that contain miRNA-targeting sites evidenced by the fact that 6mA sites are enriched at the miRNA-binding sites of target mRNAs in mouse pluripotency cells and differentiated cells [118].

### 6.3. Regulation of Long Noncoding RNA (lncRNA) by m6A Methylation

The functional secret behind the significantly high abundance of m6A in the eukaryote lncRNAs relative to other RNA molecules [41, 107, 119] has not yet been unveiled until recently, and the inverse correlation between m6A methylation levels in lnc-XIST and its silencing function was discovered [120]. As a long noncoding RNA X-inactive specific transcript, XIST functions as a gene silencer on the X chromosome at the transcriptional level. One of the m6A readers, YTHDC1, preferentially binds to m6A markers on XIST and is indispensable for XIST-conferred transcriptional silencing in human cells [121–123].

### 6.4. Regulation of m6A on circRNAs

Circular RNAs (circRNAs) belong to a new type of ncRNAs bearing the covalently closed-loop structures and universally expressed in lower and higher eukaryotes [124]. While their functions remain largely elusive, emerging data suggest that circRNAs could regulate gene expression [125, 126] and are pathologically involved in the progression of some diseases, such as cancer [127] and neurological disorders [128]. A recent study showed that endogenous circRNAs may generate proteins, expanding a novel mode of cap-independent translation [129]. Recently, Zhou et al. have identified widespread m6A modifications in circRNA by genome-wide mapping of m6A sites [116, 130]. It turns out that m6As in circRNAs share the same writer and reader protein complexes with those in mRNAs, while significant distinctions exist between many m6A sites in circRNA and those in mRNAs. One of those distinctions is in the way m6A circRNAs are generated from unmethylated exons in mRNAs, and circRNAs derived from m6A-methylated exons tend to be unstable mediated by YTHDF2, suggesting that m6A modification directed the regulation of circRNAs.

### 6.5. For tRNA Methylation

tRNA serves as a key component of protein synthesis machinery. Among the heavy modifications in tRNA, presence of m6A has been confirmed, and the dynamic regulation of m6A in tRNA critically impacts its functions as well. Mammalian ALKBH1, in addition to its function as demethylator of the DNA m6dA, has been also tested to be a tRNA demethylase for demethylation of N1-methyladenosine (m1A). Enhanced expression of ALKBH1 leads to attenuated translation initiation due to demethylation of the target tRNAs, therefore giving rise to a decrease in the usage of tRNAs for protein synthesis. The dynamic regulation of the tRNA m6A is in a glucose availability-dependent manner, altogether suggesting that dynamic m6A in tRNA regulates gene expression posttranscriptionally [131].

### 6.6. For DNA Damage Response

More recently, it was reported that RNA m6A modification could regulate UV-induced DNA damage response by rapidly recruiting Pol K, a DNA polymerase implicated in DNA damage repair, to the damage sites for quick repair to confer cell survival [130].

### 6.7. Phenotypical Correlation with m6A Alterations

Phenotypically, m6A is involved in the regulation of sex determination [132, 133], male infertility [95, 134], circadian clock [135], neurological disorders [132, 133, 136], and other diseases, such as cancer [113, 137–141].

## 7. Potential Functions of DNA m6dA Modification

The development of the restriction-modification (R-M) system conferred by the abundance of m6dA in prokaryotes such as *E. coli* [38] has been unanimously acknowledged. The potential function of m6dA, although progress has been made such as that bacterial DNA m6dA could lead to differentiation of mammalian tumor cells [142], remains largely to be elusive. Dynamic alteration of m6dA in genomic DNA was associated with brain functions ([42, 136], Yao et al., unpublished data), embryogenesis [131], reproduction [40, 131], and ES cell development [47] in a range of organisms, suggesting the fundamentally biological functions of m6dA in eukaryotes besides affecting protein-DNA interaction in eukaryotes [143–145], rather than the R-M system as in prokaryotes.

### 7.1. DNA m6dA-Mediated Chromatin Remodeling

The functions of m6dA are thought to be via the m6dA-mediated regulation at chromatin structural and transcriptional levels. It has been shown that m6dAs are distributed in the linker DNA regions of H2A variant-containing well-positioned nucleosomes, such as the H2A.X deposition region in mouse ES cells [47] and H2A.Z in *Tetrahymena* [54]. This discovery suggests the function of m6dA in chromatin remodeling. In addition, some m6dA sites have high affinity with Pol II-transcribed genes, enhancing the transcription of these genes [54].

### 7.2. DNA m6dA-Mediated Dual Functions of Gene Expression

m6dA reader proteins have not yet been identified and characterized so far. Similar to MeCP2, by binding to m6dA-distributed regions, m6dA readers may recruit partners to remodel the chromatin structure. However, in contrast to 5-mC-mediated transcription silencing, m6dA confers both transcriptional activation and repression depending on the organisms and the tissues or developmental stages even on the same organism [36, 39, 40, 52, 54].

Studies showed m6dA-conferred transcription repression like the 5-mC regulation manner in many organisms ([36, 40, 42, 146]). In *Drosophila*, 6mA levels are inversely correlated with the transposon expression in the ovary [40]. In mouse, the significantly increased level of m6dA following environmental stress is negatively associated with expression of a group of neuronal genes and LINE transposons [42]. Through genome-wide 6mA and transcriptome profiling, we found that 6mA may serve as a repressive epigenetic mark on a group of genes involved in neurodevelopment and neuronal functions in *Drosophila*.

In mouse ESCs, m6dA deposition is strongly biased on the evolutional age of L1 transposons. m6dA is significantly enriched at young relative to old L1 elements, positively correlating with epigenetic silencing of such L1 transposons together with their surrounding enhancers and genes in mammalian genome [47].

In contrast to the m6dA-associated repression of gene expression, m6dA accumulation activates the expression of genes in some organisms or in some specific tissues or developmental stages in *Chlamydomonas* [36] as well as early embryogenesis of zebrafish [147], fungi [52], and adult mouse brain [148]. Alternatively, compared to the nonmethylated adenine base in DNA, m6dA can decrease the binding energies of base pairs [149] and therefore destabilize DNA duplexes, facilitating m6dA-enriched regions of DNA, unwinding, or making the DNA structure more open for transcription initiation and the downstream processing [150].

## 8. Regulation of Stem Cell Fates by RNA m6A Modification

Dynamic changes of m6A sites or levels alter the m6A landscape in epitranscriptomes of stem cells. This could lead to the enhanced or impeded expression of the key genes responsible for proliferation, differentiation, or specification during the embryogenesis and normal development of tissues/organs/individual organisms. Consequently, the fates of the stem cells are determined. Although the exact functions of RNA m6A in stem cell regulation remain to be elusive, emerging evidences have suggested the indispensable roles of mRNA m6A in ES cells, including iPS cells, ES cells, bone marrow ES cells, blood stem cells, and neuronal stem cells [61, 111, 136, 151–154] as summarized below.

### 8.1. m6A-Mediated Regulation of Somatic Cell Reprogramming

Significant demethylation of 5-mC mainly in the promoter regions of the genes encoding some pluripotency factors such as Oct4, Nanog, Sox2, and Klf4 serves as the prerequisite during somatic cell reprogramming toward induced pluripotent stem cells (iPSCs). The demethylation is mainly catalyzed by TET, consequently leading to overexpression of the defined reprogramming factors [155]. In contrast to the inverse correlation of the DNA 5-mC methylation levels and the reprogramming efficiency during somatic cell reprogramming [155], paradoxically, the elevated mRNA methylation level of m6A enhances the efficiency [156]. This was confirmed by the fact that overexpression of METTL3 and the four Yamanaka factors (*Oct4*, *Sox2*, *Klf4*, and *c-Myc*) in mouse embryonic fibroblasts (MEFs) led to elevation of m6A abundance and dramatically promoted the number of iPSC colonies. Accordingly, downregulation of methyltransferase *METTL3* expression leading to a decreased m6A level repressed the expression of Yamanaka factors and consequently inhibited the reprogramming efficiency, altogether suggesting the essential roles of the finer-tuned regulation by combining the modifications of cytosine and adenosine at both DNA and RNA simultaneously when the cells face to the significant turning point of the life processes.

### 8.2. Regulation of Normal Hematopoietic Stem and Progenitor Cells (HSPCs) by RNA m6A Modification

Recent studies are gradually unveiling the link between RNA m6A modification and regulation of normal hematopoietic and leukemia cells as well as vertebrate embryogenesis [59, 157]. METTL3 depletion in normal human hematopoietic stem/progenitor cells (HSPCs) and leukemia cells leads to a decline in RNA m6A levels, to promotion of differentiation, and to reduction of proliferation in HSPCs and myeloid leukemia cells. Conversely, overexpression of METTL3 could reverse the phenotype conferred by METTL3 depletion [59]. Comparing with healthy HSPCs or other types of tumor cells, the expression of *METTL3* at both transcriptional and translational levels was dramatically enhanced in acute myeloid leukemia (AML) cells. Furthermore, mRNA m6A modification promotes translation of *c-MYC*, *BCL2*, and *PTEN* mRNAs in human AML cell lines. METTL3 deficiency induces the differentiation and apoptosis of human myeloid leukemia cell lines, partially being ascribed to the increased levels of phosphorylated AKT. More interestingly, METTL3 depletion delays leukemia progression in in vivo mice, altogether suggesting the potential of METTL3 as a therapeutic target for AML [59].

During zebrafish embryogenesis, dynamic mRNA m6A modification levels coordinately regulate the fate of the earliest HSPCs in endothelial-to-hematopoietic transition (EHT). Similar to human HSPCs, *mettl3*-deficient embryos, a significant decrease in m6A abundance strongly represses HSPC generation mechanistically due to the delayed YTHDF2-mediated mRNA decay of the arterial endothelial genes *notch1a* and *rhoca* [157].

### 8.3. Adult Neural Stem Cell Differentiation Regulation by m6A at the RNA Level

The RNA m6A modification levels were altered dynamically from the remarkable enrichment during early embryogenesis to a rapid drop and then maintenance of the low dose thereafter. However, the overall level of m6A remains substantially higher in heads and ovaries compared to other organs/tissues [132], suggesting the potential functions of mRNA m6A modification in the nerve and reproduction system. The mutant flies with methyltransferase deficiency reduce their lifespans and accompanied by multiple behavior defects mainly exhibited in flying and locomotion [132, 133]. This result suggests the aberrant regulation of neurological regulation associated with m6A loss. Accordingly, m6A overaccumulation in *Fto*-KO mice show postnatal neurodevelopment defect and repression of both proliferation and differentiation in adult neural stem cells [136]. Consequently, this leads to a reduced brain size and poor learning and memory. Altogether, it suggests that RNA m6A modification levels must be tightly regulated to optimal levels in accordance with the physiological conditions during embryogenesis and at the normal development stages.

### 8.4. Regulation of ES Cell Pluripotency and Differentiation by RNA m6A Modification

During embryogenesis and ES cell development, expression levels between the pluripotency factors and the differentiation factors are precisely and dynamically regulated by RNA m6A methylation. RNA m6A conferred regulation, among other epigenetic modifications, to determine the fate of ESC towards self-renewal or differentiation [154]. In mESC, the Mettl3 knockdown-caused deficiency of RNA m6A methylation leads to loss of self-renewal capability. The mechanism is the m6A methylation loss-mediated degradation of the transcripts coding for developmental regulators among a large number of others. By contrast, a conflict report is available for mESCs with Mettl3 KO in a way that RNA m6A modification loss enhances self-renewal and inhibits differentiation efficiency [151]. More studies demonstrated that chromatin-associated zinc finger protein 217 (ZFP217) could coordinate distinct epigenetic and epitranscriptomic networks to play essential roles in maintaining the pluripotency of ESC and somatic cell reprogramming by two mechanisms. The one is that ZFP217 directly regulates transcription of key pluripotency and reprogramming genes. The other is that ZFP217 sequestrates METLL3 by interacting with it to repress m6A RNA deposition in a subset of RNAs including the pluripotency and reprogramming factors such as Nanog, Sox2, Klf4, and c-Myc for their stability [55].

### 8.5. Regulation of Cancer Stem Cells by RNA m6A Modification

Cancer stem cells (CSCs) are a driving force for tumor initiation and metastasis. Exposure of breast cancer cells to hypoxia promotes demethylation of m6A in NANOG and KLF4 mRNA, leading to an increased expression of these pluripotency factors. Further study confirmed that the demethylation of m6A in these mRNAs is caused by induced expression of ZNF217 and mediated by ALKBH5; exposure to hypoxia also induces ZNF217-dependent inhibition of m6A methylation. All these inductions and enhanced demethylation are in an HIF-1*α*-dependent manner [158].

RNA m6A modification regulates generation, growth, self-renewal, and metastasis/tumorigenesis of human glioblastoma stem cells (GSC). Knockdown of METTL3, a key component of the RNA methyltransferase complex, significantly enhances GSC growth and self-renewal, caused by a dramatic decrease in m6A methylation. Further study shows the alteration of mRNA m6A distribution and the consequent mRNA expression of the genes under conditions of METTL3 or METTL14 knockdown. Inversely, overexpression of METTL3 or FTO deficiency inhibits GSC growth and self-renewal. Interestingly, FTO deficiency represses tumor progression and increases the lifespan of GSC-grafted mice substantially, suggesting FTO as a potential therapeutic target for glioblastoma [140].

## 9. Regulation of Stem Cells by DNA m6dA Methylation Modification

Although DNA m6dA methylation was discovered almost at the same time as RNA m6A methylation was, the progress in understanding the biological functions largely lags behind that in RNA m6A methylation. To date, while progress has been made in understanding stem cell regulation by RNA m6A modification, stem cell regulation from DNA m6dA modification remains to be a super mystery.

### 9.1. Insect Germline Stem Cell (GSC) Regulation

The dynamic status of DNA m6dA methylation plays essential roles during *Drosophila* embryogenesis [40]. In accordance with life processes starting from fertilization, embryogenesis, to later development, expression levels of methyltransferase (not yet identified) and DMAD, the first identified demethylase of m6dA, must be tightly regulated to maintain the appropriate levels of m6dA in the genome. Overexpression or KO/KD of DMAD leads to prenatal or postnatal lethality. It seems that m6dA could maintain the self-renewal state, while removal of m6dA by its eraser DMAD promotes GSC differentiation.

### 9.2. ESC Regulation

ALKBH1, the second identified demethylase for m6dA, functions as dioxygenase specifically removing the methyl group from histone H2A. ESC with ALKBH1 deficiency enhances pluripotency but represses differentiation particularly for neural differentiation. Further study suggests that by interaction with the core transcriptional pluripotency factors, ALKBH1 plays important roles in regulation of ESC self-renewal and differentiation [105]. More evidence came from where m6dA preferentially deposited on young L1 transposons over old L1 on X chromosomes and confers L1 silencing in ESC [47].

### 9.3. Regulation of Human Bone Marrow-Derived MSCs by m6dA

In bone marrow-derived stem cells (MSC), m6dA elevation due to ALKBH1 deficiency significantly represses differentiation of MSCs, leading to the aberrant bone phenotype [159]. Molecularly, by interacting with the promoter regions of core factors indispensable for osteoblastic differentiation including Atf4, Runx2, and Osterix, ALKBH1 removes m6dA on the promoter regions of these genes. Thus, the repression mechanism could be dissected as the increased m6dA levels at the promoter regions of these core factors in accordance with ALKBH1 deficiency, hampering the expression of these differentiation-conferring factors.

## 10. Concluding Remarks

In the recent two decades, significant achievements have been made in an epigenetic study particularly 5-mC and its intermediates such as 5-hmC, 5-fC, 5-caC, and more recently 6mA and m6dA. Introduction of bacterial m6dA-bearing DNA to mammalian tumor cell lines led to the differentiation of tumor cells [142], shedding light onto m6A modification-mediated tumor therapy. Since then, m6A study was significantly enhanced in identification of more writers, erasers, and particularly readers of m6dA modification in genomes as well as their partners for network coordination-based regulations. So far, significant achievements have been made in understanding the generation, dynamic alteration, machinery, distribution, and biological functions molecularly and phenotypically in the recent few years. However, a large number of unknown mysteries behind the RNA m6A and the DNA m6dA remain to be elusive. Since RNA m6A and DNA m6dA belong to different layers of modifications, we separately discuss about them.

So far, information on m6dA writers, erasers, and m6dA readers remains largely unknown. To better understand the functions of m6dA, it is of significance to dissect the exact mechanisms of m6dA-mediated regulations on a wider range of species. (1) Additional components of the machinery for m6dA methylation (writers)/demethylation (erasers), readers, and associated effectors need to be identified. (2) Once these machinery components are identified, their functions should be targeted at molecular, physiological, and phenotypic levels. (3) It is of importance to understand the molecular and cellular mechanisms of the deposition of m6dA in the genomes particularly in stem cell genomes. (4) 5-mC can be oxidized by TET to generate 5-hmC, 5-fC, and 5-caC intermediates, and likewise, RNA m6A could be converted by FTO to generate 6-hmA and 6-fdA as its intermediates. Thus, it is necessary to determine if m6dA could be converted into 6-hmdA, 6-fmdA, and 6-cadA as well either by TET or erasers such as FTO and ALKBH1. If it is true, their functions will be an interesting target as intermediate products for the final removal of methyl groups or as epigenetic markers for any known biological functions. It is well known that 5-hmC functions as an intermediate during demethylation of 5-mC in eukaryotes. Additionally, 5-hmC also serves as an important epigenetic marker involved in a wide range of spectra of biological pathways such as reprogramming, proliferation/differentiation, and tumorigenesis. Do h-m6A and 6-fmdA function as epigenetic markers like 5-hmC? (5) Evidences suggest the inverse correlation between m6dA levels and the complexity of eukaryotic genomes. Relative to the dominant abundance and the significant epigenetic regulation roles of 5-mC in vertebrate genomes, it is paradoxical so far how the 102- to 103-fold lower levels of the m6dA marker still play important roles in proliferation and differentiation of mammalian ESCs. [160] suggest the temporal or spatial distribution of m6dA to serve as a complementary and alternative DNA marker instead of being relatively constitutive from the generation/disposition to the functions. Since the extremely low dosages of m6dA in higher eukaryote mammals do not seem to come from relics during the evolution from prokaryotes to eukaryotes, it remains to be an interesting and essential issue for understanding the different layers of epigenetic regulations. (6) Loss of DNA 5-hmC has become a hall marker for cancer cells. Likewise, compared to the adjacent normal tissue, significant m6dA loss in human primary tumors has been detected (unpublished data). Thus, it is of great importance to compare the levels of 6mA in a variety of tumors to confirm if loss of 6mA modification could be a novel hallmark of cancers for epigenetic diagnostics of cancers or other diseases. (7) Furthermore, some compounds that could induce DNA damages have been acknowledged to play essential roles in cancer therapies. Overexpression of ALKBH family members in some cancers, such as bladder, prostate, and pancreatic cancers, inhibits cancer DNA damage, leading to cancer cell proliferation and chemotherapy resistance [161, 162]. Thus, efforts were worthy of being paid to study whether or not ALKBH family members such as ALKBH1 could serve as therapeutic target(s) for clinical cancer therapy.

For RNA m6A modification, although significant progress has been made in recent years, significant challenges remain. (1) More components in the processing machinery complexes for m6A methylation or demethylation may exist and wait to be identified. Discovery of more components could help us understand the regulation of the dynamic alteration of m6A levels. ZFP217 is the first modifier that could coordinate the distinct epigenetic and epitranscriptomic networks to maintain the pluripotency of ESCs and somatic cell reprogramming. It will be of great significance to further identify and characterize more coordinators/modifiers that could directly regulate transcription of key regulation genes. Simultaneously, the potential coordinators/modifiers could interact with m6A RNA methylation/demethylation machinery complexes. Consequently, these modifiers/coordinators could regulate the transcription and m6A RNA disposition in a subset of RNAs including the factors indispensable for pluripotency, differentiation, and reprogramming, and other key metabolic pathways. (2) More precise techniques are required to analyze the exact distribution of m6A in epitranscriptomics from different organisms. Since the exact molecular mechanisms of the selection of mRNA targets and the m6A sites in the targets remain largely unknown, efforts should be made for determination. (3) Further identification of m6A readers and repellers and characterization of their functions will help us understand the m6A-based epitranscriptomic regulation of the wide spectrum of biological processes. (4) Given that the known m6A RNA demethylases ALKBH1 and FTO could catalyze demethylation of m6A in both RNA (mRNA and tRNA) and DNA, it is of importance to investigate if other major components in the m6A methylase complex machinery such as METLL3–METTL4 could function as m6dA writers for DNA modification as well. (5) It has been identified that there are 6-hmA and 6-fmA during demethylation of m6A in the RNA, but their functions remain to be elusive. (6) It is also of importance to test if mammalian TET family members could catalyze the demethylation of m6A and m6dA in both DNA and RNA modifications, although these members did not show the demethylation activity of m6A RNA in our lab. (7) METTL3 depletion delays leukemia progression in in vivo mice, shedding light on the potential of METTL3 as a therapeutic target for human AML [59]. Thus, further exploring therapeutic targets involved in m6A machinery complexes might be very promising for some stubborn diseases such as cancers and neurological diseases. These extensive studies may unveil more exact mechanisms and the regulation roles in multiple biological processes.

## Figures and Tables

**Table 1:** Machinery of adenosine methylation in DNA and RNA.

Machinery	Component	Roles in the complex	Localization	Organism	Biological function
Writers for m6dA	DAMT-1	Methylation of DNA adenosine	Nuclei	*C. elegans*	

Erasers for m6dA	DMAD	Demethylation of m6dA	Nuclei	*Drosophila*	
	NMAD-1	Demethylation of m6dA	Nuclei	*C. elegans*	
	ALKBH1	Demethylation of m6dA and tRNA	Nuclei/cytoplasm	Mammals	
	FTO	Demethylation of m6dA and RNA m6A	Nuclei/cytoplasm	Mammals	

Readers for m6dA	Unknown	Unknown			

Writers for m6A	METTL3	m6A methyltransferase	Nuclei		
	METTL14	Core component of the m6A methyltransferase in human	Nuclei		
	MT-A70	Complex	Nuclei		
	WTAP	Regulatory component of the complex	Nuclei		
	KIAA1429	Regulatory component of the complex	Nuclei		

Erasers for m6A	ALKBH5	m6A demethylase	Cytoplasm	Mammals	mRNA export and RNA metabolism
	FTO	m6A demethylase	Cytoplasm	Mammals	Mammal fertility convert m6A to h-m6A, f-m6A

Readers for m6A	YTH family				
	(i) YTHDF1	m6A reader	Cytoplasm	Mammals	Trigger/enhance translation of mRNA bearing m6A
	(ii) YTHDF2	m6A reader	Cytoplasm		mRNA decay
					Mouse female fertility
					cap-independent
					Translation in nuclear
	(iii) YTHDF3	m6A reader	Cytoplasm	Mammals	Concordance of YTHDF1 and YTHDF2
	(iv) YTHDC1	m6A reader	Nuclei	Mammals	Facilitate inclusion of alternative exons
	(v) YTHDC2	m6A reader	Cytoplasm	Mammals	Fertility of mouse
	hnRNP family				
	(i) hnRNP-A2/B1	m6A reader	Cytoplasm	Mammals	Alternative splicing processing of miRNA
	(ii) hnRNP-C	m6A reader	Cytoplasm	Mammals	Alternative splicing processing of miRNA
	(iii) hnRNP-G	m6A reader	Cytoplasm	Mammals	
	(iv) hnRNP-H1	m6A reader	Cytoplasm	Mammals	
	(v) hnRNP-H2	m6A reader	Cytoplasm	Mammals	
	KH/RM/RBD family				
	(i) FMR1	m6A reader	Cytoplasm/nuclei mammals		FMR1/YTHDF1 share overlapping mRNA targets
	(ii) FXR1	m6A reader	Cytoplasm/nuclei		
	(iii) FXR2	m6A reader	Cytoplasm/nuclei		
	(iv) KHSRP	m6A reader	Cytoplasm/nuclei		

Repellers for RNA m6A	(i) G3BP1	Repel to m6A	Cytoplasm/nuclei mammals		Positively affect stability of their target mRNAs
	(ii) G3BP2	Repel to m6A	Cytoplasm/nuclei		
	(iii) CAPRINI	Repel to m6A	Cytoplasm/nuclei		
	(iv) USP10	Repel to m6A	Cytoplasm/nuclei		
	(v) METTL16	Repel to m6A	Cytoplasm/nuclei		

**Table 2:** Distribution of the methylated adenosine in DNA and RNA.

Species	DNA m6dA distribution	Functions
*D. melanogaster*	Transposons, intergenic regions, nucleosomal biased, preferential for repeat sequences	Promotion of transposon expression
		Repression of many genes involved in CNS functions
		Promote GSC differentiation

*C. reinhardtii*	TSS of more than 14,000 genes actively	Mainly promote gene transcription
	Linker DNA biased	
	Intergenic regions	

*C. elegans*	No preference in the genome	Mainly promote gene transcription
	Nucleosomal biased	

*T. thermophilus*	5′ end of the gene body	Enhance transcription of the genes bearing m6dA sites
	AT motif of the linker DNA regions flanked by nucleosomes particularly	
	H2A.Z-containing nucleosomes associated with Pol II-transcribed genes	

*Danio rerio*	Preferential for repeat sequences	

*Xenio laevis*	Depleted at TSSs	

*Mus musculus*	Depleted at TSSs, enriched on LINE-1 in ESC	Epigenetic silencing of LINE-1 and surrounding
	Varies in accordance with physiological conditions	Enhancers and genes
		Involved in ESC self-renewal and differentiation

*Homo sapiens*	Depleted at TSSs, enriched on LINE-1	Similar to mouse

Species	RNA m6A	Biological functions
Mammals	5′UTR and 3′UTR	Regulation of gene expression
	Stop codon	RNA metabolism including mRNA, rRNA, tRNA, miRNA, snoRNA, and circRNA
	Low abundance in coding regions long introns	
		Determination of cell fate
